# Preparation of Polystyrene Nanoparticles with Environmental Relevance Using a Gradual Degradation Method

**DOI:** 10.3390/polym17121715

**Published:** 2025-06-19

**Authors:** Hisayuki Nakatani, Mika Asano, Masaki Sakamoto, Suguru Motokucho, Anh Thi Ngoc Dao, Hee-Jin Kim, Mitsuharu Yagi, Yusaku Kyozuka

**Affiliations:** 1 Chemistry and Materials Engineering Program, Graduate School of Integrated Science and Technology, Nagasaki University, 1-14 Bunkyo-machi, Nagasaki 852-8521, Japan; bb54124102@ms.nagasaki-u.ac.jp (M.A.); bb54125111@ms.nagasaki-u.ac.jp (M.S.); motoku@nagasaki-u.ac.jp (S.M.); anh.dao@nagasaki-u.ac.jp (A.T.N.D.); 2 Organization for Marine Science and Technology, Nagasaki University, 1-14 Bunkyo-machi, Nagasaki 852-8521, Japan; kyozuka@nagasaki-u.ac.jp; 3 Fisheries Bioresources Program, Graduate School of Integrated Science and Technology, Nagasaki University, 1-14 Bunkyo-machi, Nagasaki 852-8521, Japan; heejin@nagasaki-u.ac.jp (H.-J.K.); yagi-m@nagasaki-u.ac.jp (M.Y.)

**Keywords:** microplastics, mechanism, sea, polystyrene, degradation method, environmental relevance

## Abstract

This study investigates the environmental degradation of polystyrene (PS) microparticles and flakes using a gradual degradation method. The concentration of SO_4_•^−^ decreased exponentially, simulating the environmental conditions. The nanofragment size of PS particles evolved dynamically, fluctuating from below 250 nm at 3 days to 300–500 nm at 6 days, then forming two peaks below 200 nm at 9 days, before shifting to a single peak below 100 nm at 12 days. At 15 days, the distribution expanded to two peaks between 500 nm and 200 nm. The polydispersity index (PDI) varied unpredictably, and fragments below 100 nm fluctuated between 10 and 50 nm independent of time. SEM analysis revealed an initial peeling process, with the outermost layer peeling off. The core size of the PS particles decreased rapidly from 11,000 nm to 2500 nm within 6 days and stabilized at 1000 nm after 9 days. The PS flakes showed minimal shape change until 24 days, but surface roughness increased by 30 days, leading to fragmentation. By 42 days, the flakes partially broke into ca. 100 μm pieces. The initial morphology significantly influenced the breakdown pattern, suggesting multiple breakdown mechanisms other than peeling.

## 1. Introduction

The world’s oceans are polluted with plastic [1,2,3,4,5]. When plastic products are thrown into the environment as waste, they degrade under the sun’s rays and due to mechanical stress from wind and waves, breaking down into microplastics (MP) smaller than 5 mm [6,7] and then further into nanoplastics (NP) smaller than 1000 nm [8,9]. The recovery and confirmation of NPs in the environment require extremely precise handling [10]. Nevertheless, some studies have confirmed the presence of NP particles in the oceans [8,9], raising concerns due to their unprecedented stability and unknown effects on human health. Research on NPs has primarily focused on polystyrene (PS) particles and mechanically ground commodity plastics [11,12,13]. One study has examined the ecological impact of NPs, which can interfere with lung surfactant function [12]. PS-NPs have shown reproductive toxicity in animal models, which is linked to oxidative stress and mitochondrial dysfunction, potentially affecting human reproduction [13]. NPs are found in various environmental matrices, including air, water, soil, and food, and they can enter the human body through inhalation, ingestion, or dermal absorption [12,14]. They may reach the brain, potentially causing diseases by initiating molecular or cellular reactions, damaging the blood–brain barrier, inducing oxidative stress and inflammatory responses, affecting acetylcholinesterase activity, causing mitochondrial dysfunction, and interfering with autophagy [15]. These findings highlight the significant health risks posed by NPs and underscore the need for further research and awareness [12,13,14,15].

To clearly demonstrate the actual impact of NPs on living organisms, including humans, it is essential to establish a method for creating NP models with environmental relevance [9]. Actual NPs are mainly composed of fragments of plastic waste degraded by exposure to sunlight and depend on the amount of industrial plastic produced. Therefore, it is necessary to generate NP models by fragmenting polypropylene (PP), polyethylene (PE), and polystyrene (PS). Considering the environmentally related mechanistic aspects of degradation, it is necessary to use autoxidation degradation [16,17]. A method using UV light or a xenon lamp has often been used as a method of micronization by autoxidation [18,19]. Certainly, since it is sunlight that initiates autoxidation in nature, the use of light to induce autoxidation is environmentally relevant. On the other hand, conventional photo-induced autoxidation is continuous, where light is irradiated to cause degradation at a constant rate. This is because degradation by light is slow, and acceleration is required to achieve the desired effect. The constant exposure to light is very different from natural degradation. The sun sets, and the intensity of sunlight varies with the seasons. Sunlight is also blocked by biofilm and dust. A particularly important consideration is ocean degradation. Plastic debris slowly sinks into the ocean, and the intensity of degradation by sunlight gradually decreases. There should be a difference in the fragmentation of plastics between such “uniform degradation” and “gradual degradation”, but no one has yet investigated what kind of fragmentation behavior occurs in plastics subjected to “gradual degradation”. Controlling autoxidation degradation is difficult, but it is possible if the stability of the hydroperoxide group, which is the rate-limiting step, and the generation of active oxygen, which is the initiating species, can be controlled [20,21]. In our previous study [22], we conducted PP degradation in seawater using an advanced oxidation process (AOP) with sulfate ion radical (SO_4_•^−^) as a highly efficient initiator for plastic degradation. The combination of seawater and the SO_4_•^−^ initiator resulted in the excellent acceleration of the degradation process under pH control. This accelerated degradation method can be applied not only to PP, but also to PS, which has the same autoxidation degradation mechanism [23]. Therefore, we believe that it is possible to reproduce the “gradual degradation” with some degree of acceleration.

In this study, the gradual degradation behavior of PS microparticles under environmental conditions was investigated while preventing aggregation during the degradation using Triton^®^ X-114 surfactant. Particle size fluctuations were measured at various intervals, and complex changes in the size distribution of PS nanofragments were studied. The breaking down mechanism of PS microparticles was estimated by analyzing scanning electron microscope (SEM) images. In addition, by using PS flake samples as a model for marine plastic waste, gradual degradation was performed, and the degradation process was studied. The effect of sample shape on fragmentation was assessed by comparing the behavior of PS microparticles and flakes in detail. This study is the first to demonstrate its application to PS in a simulated marine environment with a focus on environmentally relevant degradation behavior. Our experimental design introduces a novel approach to simulate real-world environmental conditions by controlling the concentration of SO₄•⁻ to mimic the attenuation of sunlight due to natural factors such as cloud cover, diurnal cycles, and particle sinking.

## 2. Materials and Methods

### 2.1. Materials

PS particles (Standard Particle PHARM-TROLTM Series 15 μm) were obtained from Thermo Fisher Scientific K.K. (Tokyo, Japan) with a size of ca. 15 μm, and their particle count was 3800 ± 15%/mL. Wako Pure Chemical Industries (Osaka, Japan) provided potassium persulfate (K_2_S_2_O_8_), PS pellets (the weight-average molecular weight and molecular weight distribution were 3.5 × 10^5^ and 2.1, respectively.), and Triton(R) X-114 (Polyethylene glycol tert-octylphenyl ether). The seawater used was artificial (Gex artificial saltwater), which was purchased form Amazon.co.jp (Tokyo, Japan. https://www.amazon.co.jp/-/en/GEX-Gex-Seawater-6-5-Pieces/dp/B09R9X4Y43?th=1, accessed on 5 September 2024).

### 2.2. Pulverization and Film Molding Methods Using PS Pellets

The PS pellet pulverization was carried out with a freeze crusher (TPH-01, AS ONE Co., Osaka, Japan) with liquid nitrogen to obtain the powder. The powder was passed through a sieve with 100 mesh (Filtration particle size: ca. 250 μm) and was employed as a pulverized sample. The specimen for PS film was ca. 30 mm × 30 mm × 0.075 mm. The film was obtained by compression molding at 180 °C under 10 MPa for 11 min.

### 2.3. Gradual Degradation Using Sulfate Ion Radicals in Seawater

The gradual degradation of the PS particles was carried out by means of an advanced oxidation process (AOP) with sulfate ion radicals in the artificial seawater. The procedure was in accordance with our previous reports [22,23]. (1) The 25 mL PS particle solution (containing a total of approximately 95,000 ± 15% particles) was put into a 100 mL glass vessel containing 20 mL of seawater solution with 0.54 g K_2_S_2_O_8_ at ca 65 °C for 12 h under stirring with a stirrer tip speed of ca 100 rpm. (2) To compensate for the consumption of the oxidizing agent, 20 mL of the same K_2_S_2_O_8_ seawater solution was added, and the degradation was carried out for 12 h under the same conditions. (3) As the 20 mL K_2_S_2_O_8_ seawater solution was added every 12 h, the volume of the solution continued to increase; thus, the entire solution was transferred to a larger vessel as appropriate. The degradations started again under the same conditions. All gradual degradation was carried out at the same temperature (65 °C) and with a stirring speed of 100 rpm. The pH value of the solution was changed from 8.2 to 3 during each set (the pH of the seawater was initially 8.2, and the SO_4_•^−^ was gradually converted to SO_4_^2−^, reducing the pH of the seawater solution to 3 at the time of the daily exchange [22,23]). The samples that had reached the predetermined degradation time were filtered using filter paper with a pore size of 200 nm, rinsed with a small amount of pure water, and then dried in a vacuum dryer at 60 °C for 7 h before being used for each measurement.

The gradual degradation of the 10 mg pulverized PS pellet was first put into a 100 mL glass vessel containing 20 mL of seawater solution with 0.54 g K_2_S_2_O_8_ at ca 65 °C for 12 h under stirring with a stirrer tip speed of ca 100 rpm, and then the same procedure as in the gradual degradation method of PS particles was used. The filtration and drying methods were also the same as those used for the PS particles.

### 2.4. Characterization and Analysis

Scanning electron microscopy (SEM) analysis was conducted using a JSM-7500FAM (JEOL, Tokyo, Japan) at an electron beam voltage of 5.0 kV. The working distance was approximately 3 × 4 mm. The samples were placed in a drying oven maintained at 27 °C for 30 min and sputter-coated with gold before undergoing SEM imaging.

The hydrodynamic size was determined under pure water suspension at 20 °C using an ELSZ-2000ZS dynamic light scattering (DLS) analyzer manufactured by Otsuka Electronics (Osaka, Japan). The hydrodynamic size of the fragment contained in the conical tube after processing was determined by measuring the path length and width of a borosilicate glass standard fluorescence cell (10 mm each) and the capacity (3.5 mL). The DLS analyzer was used to measure the particle size distribution. The range of sizes was measured from 0.6 nm to ca. 15 µm, with the DLS analyzer operating at a temperature of 20 °C and using pure water (viscosity 0.8878 cP, refractive index 1.3328) as the solvent for each measurement. The DLS analyzer performed 25 accumulations for each sample.

The transform infrared spectra of 16 scans were measured with a Fourier transform infrared spectrometer Jasco FT-IR 660 plus (Jasco, Tokyo, Japan) with a resolution of 4 cm^−1^ over the entire mid-IR range (400–4000 cm^−1^).

### 2.5. Statistical Processing

Hydrodynamic size distribution and polydispersity index (PDI) were calculated using a software (ELSZ-2000 Version 7.16, Otsuka Electronics Co., Ltd., Osaka, Japan) supplied with the DLS.

## 3. Results and Discussion

### 3.1. Peeling and Breaking Down Behavior of PS Particles by Gradual Degradation

**Figure 1:**
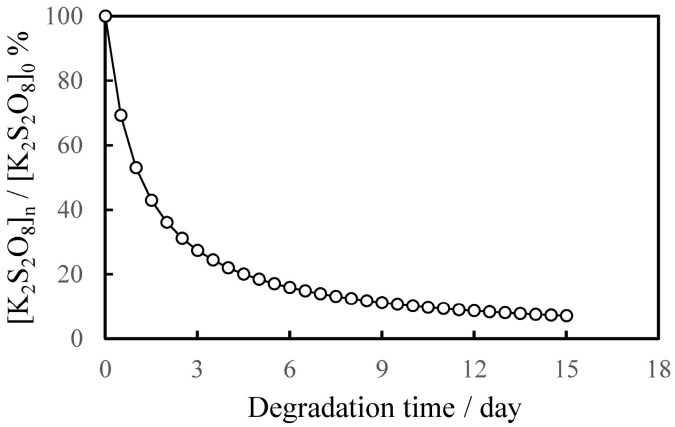
Dependence of degradation time on K_2_S_2_O_8_ concentration ratio: [K_2_S_2_O_8_]_0_ = 44.4 × 10^−3^ mmol/L.

**Figure 2:**
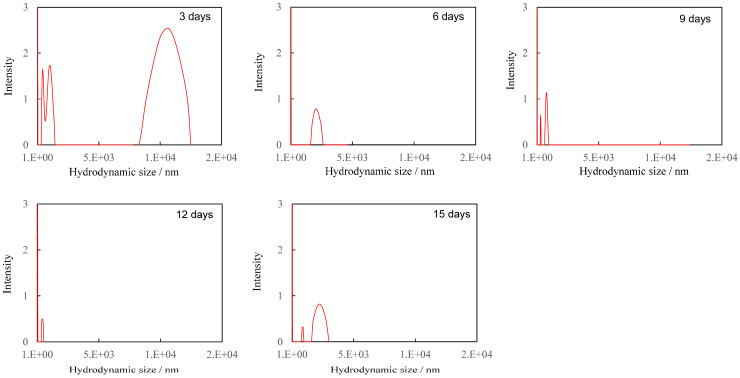
Hydrodynamic size distribution of all PS particles at each degradation time.

As shown in Figure 1, the concentration of K_2_S_2_O_8_, i.e., SO_4_•^−^ decreased exponentially with the increasing degradation time. By controlling the concentration of SO_4_•^−^ in this way, we were able to successfully observe the gradual degradation behavior of PS particles by reducing the degradation rate as the degradation time increased. This gradual degradation behavior simulated environmental degradation due to the attenuation of sunlight caused by factors such as changing day/night, sunlight being blocked by clouds, and the sinking of irradiated targets to the seafloor. Figure 2 shows the hydrodynamic size distribution of all PS particles at each degradation time. The hydrodynamic size of degraded PS particles shows complex distribution behavior at each degradation time. After 3 days of degradation, there were particles close to the original particle size of 8 to 15 μm, but after the 6 days, they decreased to 3 μm. After 6 days of degradation, relatively large particles of a few microns did not show a clear size dependence on degradation time, but instead they showed repeated size increases and decreases. The peak on the left of the graph for each degradation time indicates particles of several hundred nanometers, which have a complex size distribution. For degradation times of 3 and 9 days, the corresponding peaks appear as double peaks, while for other degradation times, the peaks appear as single peaks. When measuring DLS, 1% of the surfactant [Triton(R) X-114] was added. In the previous research [23], the same concentration of Triton(R) X-114 surfactant was successful in suppressing the formation of aggregation structures in low-density polyethylene (PE) nanoparticle fragments produced by similar AOP degradation. According to the report by Li et al. [24], the most important factor for the nano-dispersibility of PS nanoparticles is the charge on the surface of the nanoparticles. The charge on the surface of the nanoparticles is negative and is derived from oxidation compounds containing carbonyl groups, etc., which are produced by decomposition. The longer the degradation time, the greater the absolute value of the negative charge is expected to be. Increasing the absolute value of the negative charge on the surface increases the repulsive force between nanoparticles, making them less likely to aggregate. Therefore, the use of surfactants is essential in cases where the degree of degradation is low. In this study, the use of Triton(R) X-114 surfactant is essential to suppress the aggregation of PS fragments because the ability to degrade PS decreases over time. As shown in Figure 2, the number of PS particles in the hundreds of nanometer size range continues to increase and decrease as the degradation time progresses. For example, at a degradation time of 3 days, the particle size distribution showed two splits at 250 nm or less for the relevant particle size, but at a degradation time of 6 days, it showed a slightly broad monodisperse particle size distribution from 300 nm to 500 nm. After the degradation time of 9 days, the particle size distribution showed two peaks of less than 200 nm, and after 12 days it further decreased to a single peak of less than 100 nm. After 15 days, it increased again to a two-peak distribution from 500 nm to 200 nm.

**Figure 3:**
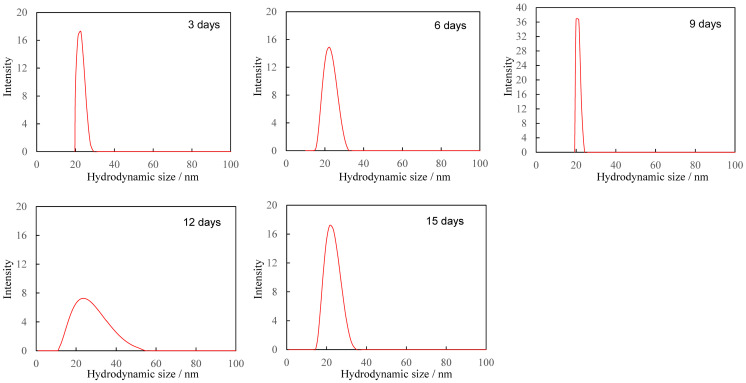
Hydrodynamic size distribution of particle fragments smaller than 100 nm at each degradation time.

**Figure 4:**
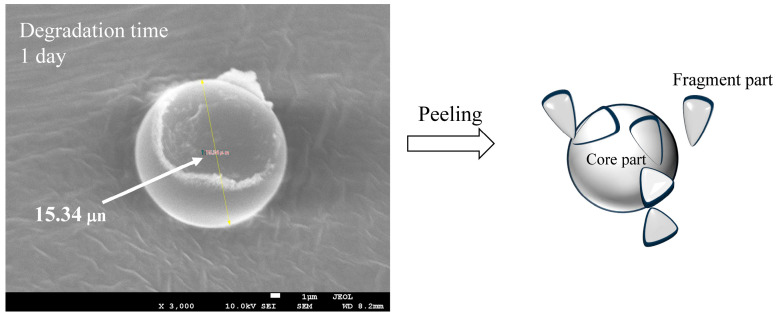
SEM image of a gradually degraded PS particle and image of peeling.

**Figure 5:**
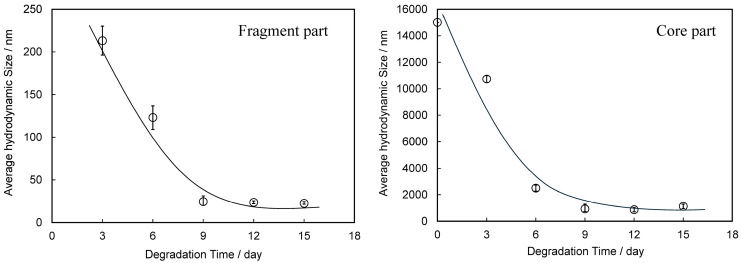
Degradation time dependences of average hydrodynamic size in fragment and core parts.

**Figure 6:**
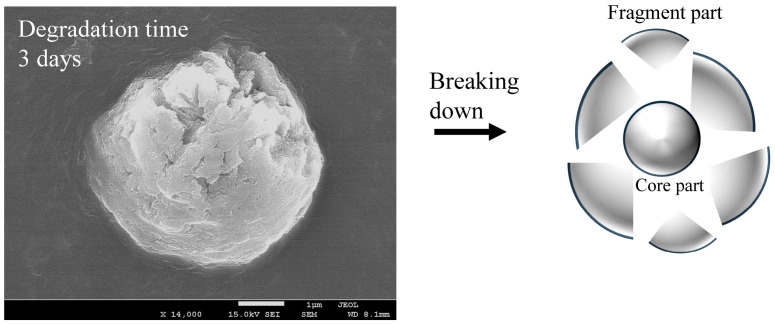
SEM image of a gradually degraded PS particle and image of breakdown.

The dependence of PDI on degradation time is summarized in Table 1. The PDI value repeatedly increased and decreased with degradation time, but no clear dependence could be observed. Such complex changes in particle size distribution over time suggest that new fine fragments of different sizes are being created over time, or that further refinement is occurring, resulting in fragments that are too small to measure. Figure 3 shows the hydrodynamic size distribution of fragments smaller than 100 nm at each degradation time. Even in the smaller nanoscale region, the size of the PS particle fragments did not show any dependence on degradation time, as was the case for the larger size, and the width of the size distribution repeatedly increased and decreased from 10 nm to 50 nm. These changes in size distribution and PDI are independent of degradation time and are therefore thought to be related to the disappearance of fragments that reached the measurement limit due to the generation of new fine fragments by further fragmentation. In fact, the change in particle fragmentation size distribution is not related to degradation time, suggesting that the degree of degradation in the fragments does not depend on the degradation time. It is necessary to accept the behavior that when degradation progresses to a certain degree, the surface layer peels off and a new, less degraded surface appears in order to explain the change in particle size distribution. Some research groups have reported that the photodegradation of polyolefins causes the degrading layer to detach from the surface [25,26]. Figure 4 shows the SEM image of PS particles degraded for 1 day, schematically illustrating the peeling from the surface layer. It can be observed that it is starting to peel off. The gradual degradation causes the core and fragment parts. The SEM photograph captures the initial stage of the peeling process, showing that the outermost layer appears to be peeling away. The diameter of PS particles immediately after peeling was 15.34 μm, which was almost the same as the diameter before the gradual degradation. No decrease in micro size was observed in the PS particle before and after the gradual degradation, i.e., before and after the peeling away. The fact that the size distribution and PDI of fragments do not depend on degradation time can be explained by the decrease in degradation rate due to radical initiators, which decrease exponentially with increasing degradation time. In fact, as shown in Figure 5, when the fragments are divided into a fragment part (1000 nm less) and a core part (1000 nm or more), the plot of the average fragment size (average hydrodynamic size) of the fragment part versus degradation time shows an exponential decreasing trend versus degradation time. After the degradation time of 3 days, the average hydrodynamic size of the fragment part was ca. 200 nm, and after 9 days, it decreased to ca. 20 nm. However, it remained almost constant during the subsequent degradation time. This is because the peeling part gradually degrades and becomes finer in accordance with the decrease in SO_4_•^−^ concentration and because the newly peeling part from the core becomes smaller in response to the decrease in concentration. Interestingly, the average hydrodynamic size of the core part also decreases exponentially with degradation time. The mechanism by which nanometer-thick fragments peel off from the approximately 15 μm core part as degradation progresses cannot explain such an exponential reduction in hydrodynamic size. This is because, as the radical concentration decreases, the rate of degradation slows, making it more difficult for the material to peel off, and the fragments that do peel off become smaller and thinner. The result that the hydrodynamic size of the core part shows an exponential decrease with respect to the degradation time suggests the existence of fragmentation mechanisms other than peeling. In photo-degradation reactions, it is known that the progression of degradation slows down toward the interior due to the diffusion of oxygen [27,28]. White et al. reported that, in ultraviolet-degraded polypropylene (PP), cracks in the surface layer that has undergone degradation are arrested on reaching the ductile material in the interior, which has not been degraded or has been degraded to a low degree and retains its toughness [29]. In our degradation system using SO_4_•^−^ in seawater, oxygen permeability is similar, with the oxygen concentration decreasing from the surface to the interior of PS particles. Depth degradation is strongly influenced by the diffusion behavior of oxygen, even in degradation caused by this system. PS is an amorphous polymer, so it does not undergo chemical crystallization when it degrades and, therefore, is not affected by residual stress in the direction of cracking. The depth and density of cracks appear to be relatively uniform across the surface of the sphere. The peeling occurs in two dimensions, and the thickness of the peeling area is considerably smaller than its length in both the vertical and horizontal directions (see Figure 4). After peeling, cracks in the depth direction remain on the surface. As the SO_4_•^−^ concentration decreases, the next peeling area becomes smaller, but as the radicals penetrate from the cracks into the depth direction, the depth of the cracks gradually increases. Obviously, the penetration of radicals into the crack causes degradation not only in the depth direction, but also in the vicinity, leading to the embrittlement of the entire matrix. When the matrix exceeds a certain threshold of brittleness due to shear caused by the stirring of seawater and collisions between particles, it breaks down. As shown in Figure 5, the average hydrodynamic size of the core decreased rapidly from ca. 11,000 nm at the 3-day degradation time to ca. 2500 nm at the 6-day degradation time. This behavior can be explained by considering the breakdown mechanism. Figure 6 shows the SEM image of PS particles degraded for 3 days, schematically illustrating the breaking down. In the SEM image, the PS particle appears as an irregular sphere with defects here and there, with a diameter of ca. 5 μm. This sphere is the core part that broke into large fragments during the breakdown, which is evidence of the existence of a breakdown mechanism. As shown in Figure S1, the diameter of the core decreased from 10 μm to 1 μm as the degradation time increased up to 9 days and remained at approximately 1 μm for longer degradation times. On the other hand, the shape remained spherical, albeit irregular, regardless of the degradation time. The result that the shape was maintained suggests that a repetitive mechanism occurred in which the interior, which had been degraded to a low degree, was exposed to the surface by the breakdown, followed by further peeling and breaking down. Plastic that has weathered under natural conditions, especially in coastal areas, is known to have a distinctive pitted texture on its surface [30,31,32,33]. Plastic degradation tends to produce pit textures in marine environments where UV radiation and mechanical erosion are minimal [30]. Cooper et al. inferred that the pits observed on the surface of PE debris collected along the coast are caused by collisions [31]. In addition, although no damage was observed in other plastics, the presence of small areas of severe oxidation (which Copper et al. referred to as “initial degradation sites” and “preferential degradation”) was also noted [31]. Their information suggests that the PS particle breakdown phenomenon observed in the gradual degradation we found may complement the pit formation mechanism. Plastic weathering tests are generally performed in the air. A light source, such as a xenon lamp, is used to continuously irradiate the plastic at a constant intensity to cause degradation. Due to a number of factors, the degradation rate is expected to decrease gradually under natural conditions. In particular, the effects of seawater on coastal weathering cannot be ignored. Aqueous Cl^−^ functions as an inhibitor in the photooxidation of polymers such as polyolefin in seawater [34]. The pits observed on the surface of PE debris found on the coast are thought to have been generated by a mechanism similar to the breakdown observed in PS particles in this study. In areas of the PE debris surface that would be partially submerged in seawater, degradation due to exposure to sunlight progressed, resulting in peeling and cracking. The degradation repeatedly stopped and progressed, and cracks in the depth direction accumulated at specific locations where degradation could progress. Eventually, complex mechanical stresses such as shear forces from waves and friction with sand caused breakdowns at these specific locations, and the collapsed sections were washed away by waves, forming the pits of PE. However, whether or not a pit-like texture forms appears to depend on the type of polymer. For example, on the coast, deep and distinct crack textures are mainly observed in PP debris [31]. As described above, the PS particles disintegrated as if the outer skin had been peeled off. They retained their spherical shape. The shape of the texture is formed by degradation, and mechanical stimulation depends on the characteristics of the degraded polymer. The most important factor in determining the shape of a texture is whether it is crystalline or not. This is because crystalline polymers such as PE and PP undergo chemi-crystallization, which leads to embrittlement when they are degraded [27,28]. The depth density of cracks and the brittleness of the matrix polymer increase as degradation progresses, causing the degraded areas to fragment under mechanical stress. In this case, whether the degraded part breaks into fine particles and is removed to form pits, as in PE, or is removed in relatively large rock-like pieces to form deep, distinct crack-like textures, as in PP, would be determined by the crystallization speed and glass transition temperature of each polymer. On the other hand, in amorphous polymers such as PS, chemi-crystallization does not occur; thus, cracks in the depth direction occur at relatively uniform depths, and as degradation progresses, the density of these cracks increases, and cracks develop horizontally at a certain depth and connect two-dimensionally. Therefore, when shear forces like waves are applied, spheres, which tend to degrade at a uniform rate due to their shape, peel off in blocks of uniform thickness, and their diameter decreases in a similar manner while maintaining their shape.

### 3.2. Fragmentation Behavior of PS Pulverized Pellets by Gradual Degradation

**Figure 7:**
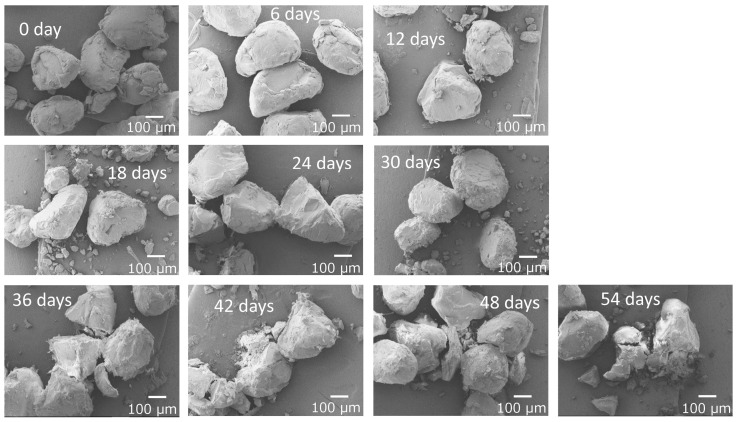
SEM photographs of gradually degraded PS pulverized pellets at various degradation times.

From an environmental impact perspective, spherical plastic waste is referred to as primary microplastics, but flake-shaped secondary microplastics generated by degradation and/or mechanical stress are clearly present in much greater quantities in the natural environment. It is possible to take spectroscopic measurements using FT-IR for flake-shaped specimens, which enables confirmation of the degradation process. Therefore, for the plastic specimens used in this study, which simulates the gradual degradation under natural conditions, flake-shaped specimens are preferable to spherical specimens. Plastic debris released into the ocean becomes covered with microalgae and other organisms, causing what is known as “biofouling”. As biofouling progresses, the surface of the debris becomes thickly covered with a biofilm, preventing sunlight from reaching the surface of the plastic debris [35]. Furthermore, as the overall weight increases, the plastic debris covered with biofouling gradually sinks into the sea. As the sunlight is gradually blocked and the plastic debris sink into the sea, the rate of degradation gradually decreases. This is exactly what is meant by “gradual degradation”. Using PS pulverized pellet flakes (major axis: a few hundred micrometers) as a model for degradation progression in the sea, the gradual degradation was used to investigate changes in shape and size by SEM observation. Confirmation of the gradual degradation (autoxidation) reaction was performed using FT-IR measurement. In the PS pulverized pellet flakes that underwent gradual degradation for 21 days, peaks assigned to carbonyl groups and hydroperoxide groups were observed at around 1720 cm^−1^ and 3500 cm^−1^, respectively, confirming that autoxidation had occurred (see Figure S2). As shown in Figure 7, no clear shape changes dependent on degradation time were observed until the 24th day of degradation. After 30 days of degradation, the flake surface became rough, and fragmentation could be observed peeling off the surface, similar to the PS particles. However, compared to the PS particles, there were significant differences in the breakdown behavior. In the PS flakes, broken pieces ca. 100 μm in size separated by crevice-like cracks were observed in samples after 42 days of degradation time. In the case of PS particles, they retained their spherical shape after the breakdown and became smaller in a similar manner, but in the case of flakes, they did not become similar in shape but rather cracked discontinuously and split into smaller pieces. The PS particles were not oriented, but the flakes retained residual stress from pulverization and were oriented in a certain direction. Therefore, cracks tended to occur in the orientation direction. The PS flakes measuring a few hundred micrometers in size were broken down into pieces measuring ca. 100 μm after 42 days of decomposition. At this time, the SO_4_•^−^ concentration had decreased to 2.6% of the original value, and the degradation intensity (speed) had significantly decreased. This indicates that the growth of crevices is not due to chemical factors but is rather strongly dependent on mechanical factors such as residual stress and shear stress from seawater. Pabortsava et al. reported that PE, PP, and PS particles with sizes ranging from 32 μm to 651 μm were suspended within the top 200 m of the Atlantic Ocean, with a total estimated quantity from 11.6 to 21.1 million tons [36]. Interestingly, the size of the fragments is similar to that of the PS pieces that broke off and separated. It is likely that suspended PE, PP, and PS particles in the sea, where sunlight does not easily reach, are generated by the same mechanical mechanism, resulting in similar sizes.

This method has great potential for use in environmental monitoring and toxicity research. In environmental monitoring, it can detect and quantify pollutants, assess ecosystem health, and track changes over time. In toxicity research, this method allows scientists to evaluate the effects of various substances on biological systems and provides insight into potential risks to humans and the environment. However, obtaining reliable and reproducible results requires considering limitations such as sensitivity and specificity and the need for standardized protocols. This requires conducting numerous preliminary experiments, which necessitates producing a large number of NP samples. Based on the mechanisms of NP formation identified in this experiment, we plan to develop methods for large-scale NP production in the future.

## 4. Conclusions

The concentration of SO_4_•^−^ decreased exponentially with degradation time, simulating environmental conditions. The size of PS spherical particle distribution changed in a complex manner. The usage of Triton(R) X-114 surfactant effectively prevented the agglomeration of fragments generated by the degradation. As degradation progressed, the nanofragment sizes fluctuated in the following way: below 250 nm at 3 day of the degradation time, 300–500 nm at 6 days, two peaks under 200 nm at 9 days, and a single peak below 100 nm at 12 days. After 15 days, a two-peak distribution reappeared, ranging from 500 nm to 200 nm. The PDI value repeatedly increased and decreased with the degradation time, but no clear dependence was observed. The fragments smaller than 100 nm, with size distribution fluctuating between 10 nm and 50 nm, did not depend on degradation time. These results suggest that new nanofragments were generated as larger fragments disappeared. The degree of degradation did not depend on time alone but rather on the peeling of the surface layer, revealing a less degraded surface. The gradual degradation formed the core and fragment parts. The SEM photograph captured the initial stage of the peeling process, showing that the outermost layer was peeling away. The fragment size distribution and PDI showed little dependence on degradation time, which was due to the exponential decrease in the radical initiator over time. After 3 days, the average hydrodynamic size of PS fragments was about 200 nm, decreasing to 20 nm after 9 days, and then remaining constant. This was due to the gradual degradation and peeling of the surface layer, influenced by decreasing SO_4_•^−^ concentration. The core size also decreased exponentially, suggesting mechanisms other than peeling, such as oxygen diffusion limiting degradation. The cracks formed uniformly, and as radicals penetrated, they caused further degradation and embrittlement. The core size rapidly decreased from 11,000 nm to 2500 nm within 6 days, indicating a breakdown mechanism. The SEM images showed irregular spheres with defects, supporting the mechanism. The core size stabilized at 1000 nm after 9 days, maintaining a spherical shape. The degradation caused peeling and cracking, with mechanical stresses leading to the breakdown. The shear forces of seawater caused the PS spheres to peel off uniformly, maintaining their shape while decreasing in diameter.

From an environmental relevance stance, the PS pulverized pellet flakes were used to simulate gradual degradation. The SEM observations showed no shape changes until 24 days of degradation time, but after 30 days, the surface became rough and fragmented. The PS flakes partially broke into pieces around 100 μm after 42 days, influenced by mechanical factors like residual stress and seawater shear stress. When the initial shape was flake-like, the breakdown shape differed greatly from that of the sphere. It was found that the shape at the time of the breakdown was greatly influenced by the shape at the start.

## Figures and Tables

**Table 1:** Dependence of polydispersity index (PDI) on degradation time.

Deg. Time	3 Days	6 Days	9 Days	12 Days	15 Days
PDI	0.246 ± 0.065	0.163 ± 0.039	0.212 ± 0.026	0.176 ± 0.038	0.154 ± 0.019

## Data Availability

Data are contained within the article.

## Supplementary Materials

The following supporting information can be downloaded at https://www.mdpi.com/article/10.3390/polym17121715/s1: Figure S1: SEM images of the core parts obtained from PS particles with gradual degradation over various degradation times. Figure S2: FT-IR spectra of gradually degraded PS pulverized pellets at 0 day and 21 days degradation times.
