# Supplementary material for: Preparation of Polystyrene Nanoparticles with Environmental Relevance Using a Gradual Degradation Method
Source: Polymers (Basel). 2025 Jun 19;17(12):1715. doi: 10.3390/polym17121715 (PMC12197181; doi:10.3390/polym17121715)
Supplement: Supplementary file 1 [file polymers-17-01715-s001.zip › polymers-3689812-supplementary.pdf]

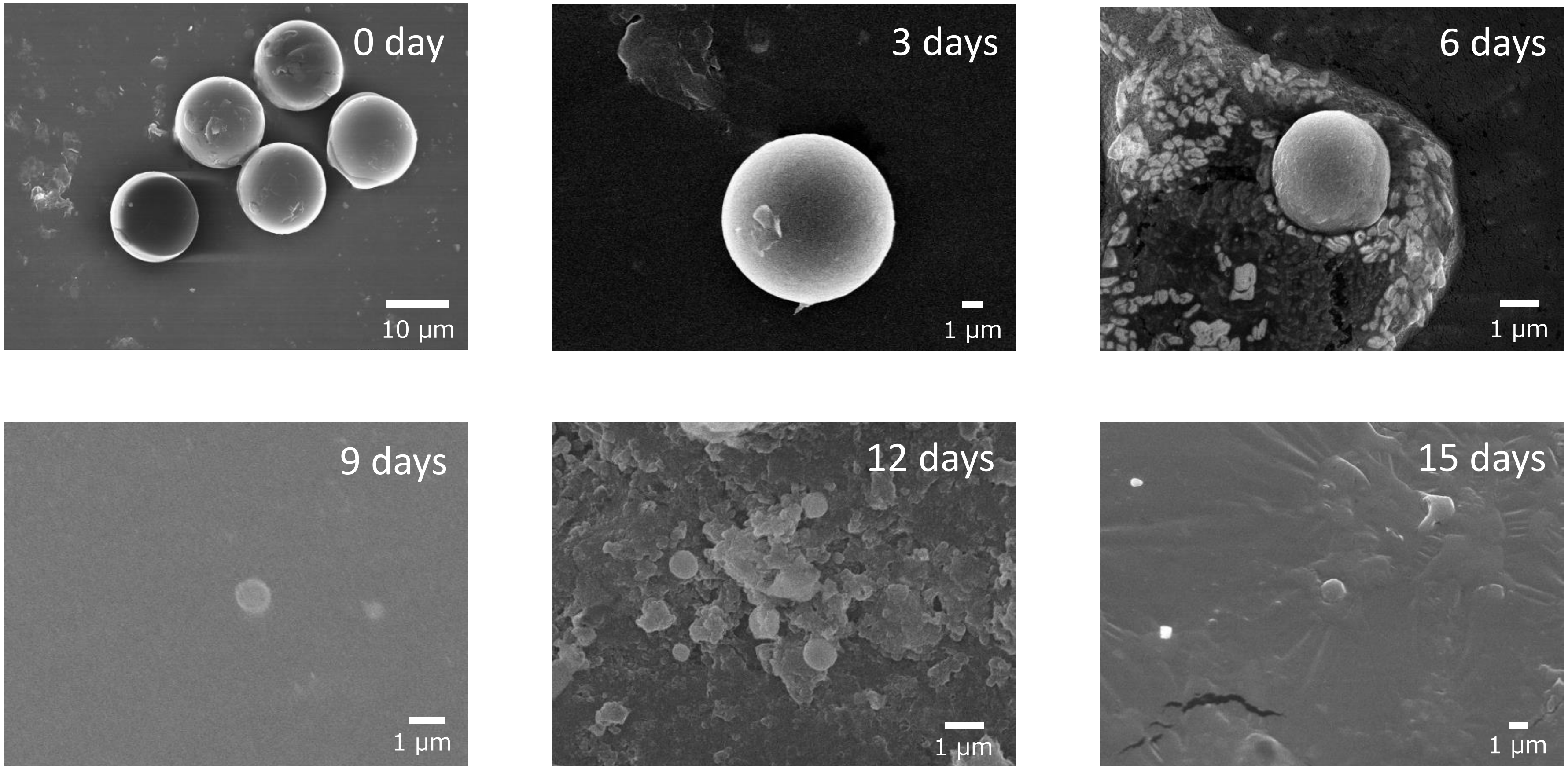

Figure S1 SEM images of the core parts obtained from PS particles with gradual degradation over various degradation times.

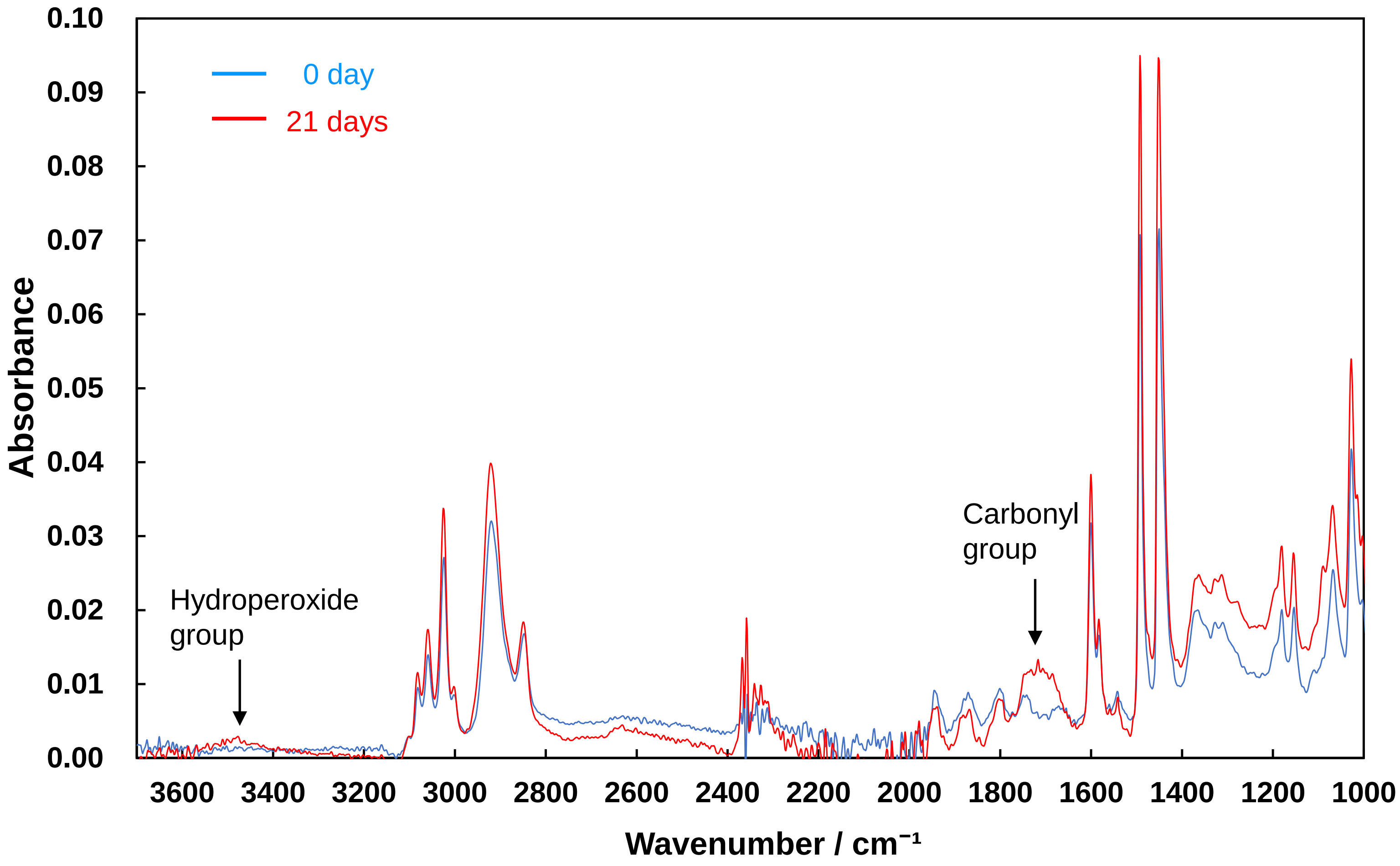

Figure S2 FT-IR spectra of gradually degraded PS pulverized pellets at 0 day and 21 days degradation times.
